# Intralesional injection of triamcinolone acetonide for cavernous hemangiomas: A case report

**DOI:** 10.1097/MD.0000000000016986

**Published:** 2019-09-13

**Authors:** Wen-yan Wang, Luan-hong Wang, Guang Huang, Zhen-ying Lin, Han Lin

**Affiliations:** a Department of Pediatric Surgery, First Affiliated Hospital of Shantou University Medical College; b Department of Gynaecological Oncology, Cancer Hospital of Shantou University Medical College; c Department of Pediatrics, First Affiliated Hospital of Shantou University Medical College, Shantou, Guangdong Province, China.

**Keywords:** buttock, cavernous hemangiomas, intralesional injection, triamcinolone acetonide (TCA)

## Abstract

**Rationale:**

Cavernous hemangiomas referred to as venous malformations (VMs), are not true vascular tumors. The treatment of cavernous hemangiomas is controversial.

**Patient concerns:**

A five-year-old girl with a cavernous hemangioma on her right buttock had undergone surgery but recurred 1 month after the operation.

**Diagnoses:**

Cavernous hemangioma was diagnosed on the basis of physical examination, magnetic resonance imaging (MRI) and postoperative pathologic examination.

**Interventions:**

We treated her with intralesional injection of triamcinolone acetonide (TCA) for 8 times.

**Outcomes:**

She was cured and had no recurrence during the 3-month follow-up.

**Lessons:**

This prompts that TCA may provide a more effective and safer choice for the treatment of cavernous hemangiomas.

## 1 Introduction

Cavernous hemangiomas, are most common in the skin and soft tissues but can be located anywhere in the body, are slow flow venous malformations (VMs).^[1,2,3]^ The treatment of cavernous hemangiomas mainly depends on surgery and venous embolization therapy. However, because of surgical risks involved in their removal, many surgeons prefer intralesional injections using long-acting glucocorticoids, such as TCA.^[4]^ We report a case of subcutaneous cavernous hemangioma of right buttock, which recurred within a month after surgery and finally recovered after subcutaneous injections of TCA in the lesion area for 8 times.

## 2 Case report

**Figure 1:**
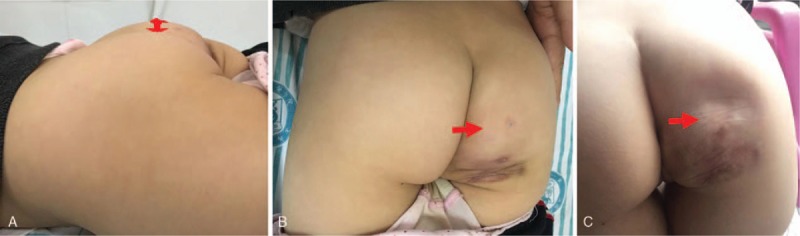
Postoperative pathology: (**A**) venous sinus of varying sizes (arrow), some of which fuse with each other (HE stain × 40). (**B**) The thin-walled (arrow) lined with endothelial cell layer (HE stain × 100). Diagnosis: “Right buttocks” consider cavernous hemangioma.

**Figure 2:**
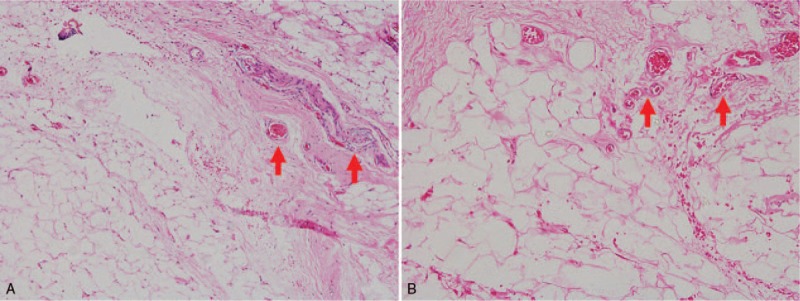
Outward appearance: (**A**) lateral view shows that the right buttock is significantly higher than the left buttock about 0.8 cm (arrow). (**B**) The skin on the surface of the right buttock is slightly red (arrow), and a 5 cm long surgical scar can be seen below. (**C**) After subcutaneous injection of TCA, hemangioma atrophy resulted in obvious depression of the right buttock (arrow).

A 5-year-old female child was admitted to our hospital, whose mother denied history of trauma to the affected area as well as any family history of soft-tissue masses. Her mother found a mass on the girl's right buttock and the right thigh for 3 days. Surgery was performed to remove the mass with a size of 5 × 5 × 3 cm. The histological study of the specimen taken intraoperatively is consistent with a diagnosis of cavernous hemangioma (Fig. 1). One month after the operation, families of the child found that her right buttock was markedly swollen compared to the left side (Fig. 2).

**Figure 3:**
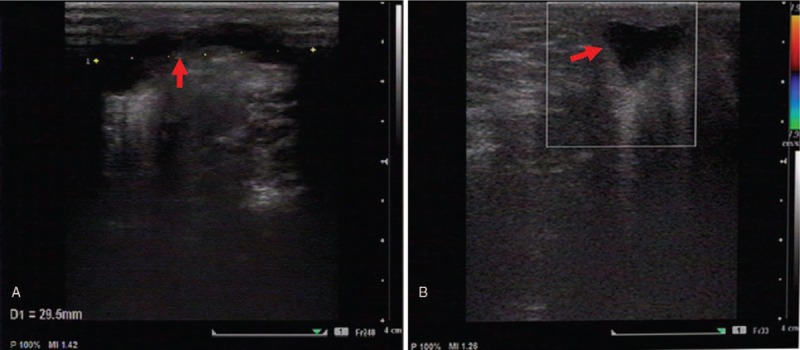
Magnetic resonance imaging (MRI) contrast before and after treatment: Before treatment, (**A**) axial T1-weighted, (**B**) axial T2-weighted, and (**C**) sagittal T2-weighted MR images show extensive exudation and irregular strip-like mixed signals (arrow) in the subcutaneous tissue of right buttock and the right thigh. After the treatment, (**D**) axial T1-weighted, (**E**) axial T2-weighted, and (**F**) sagittal T2-weighted MR images show that there was less subcutaneous soft tissue exudation in the right buttock and right thigh than before, and no obvious abnormal signal (arrow) was observed.

Physical examination showed that the right buttock is about 0.8 cm higher than the left one. The enlarging soft-tissue mass extending from right hip to right thigh was compressible, tender and nonpulsatile. The magnetic resonance imaging (MRI) showed recurrence of cavernous hemangioma (Fig. 3A–C). MRI is reserved for further clarification to confirm the extent and tissue characteristics of the lesion. The diagnosis was based on MRI and postoperative pathologic examination.

After, we informed the family of the wide range of lesions in the child and the high risk of recurrence after reoperation, the family seemingly unsuitable to have accepted surgery. It is necessary to take a more effective treatment at that time. After obtaining the informed consent from parents of the child, TCA was intralesional injected into the cavernous hemangioma under the guidance of ultrasound at a dose of 40 mg by using a 2 ml needle. The needle was inserted from the edge of the tumor, from the outside to the inside, multipoint injection. Compression with the hand was maintained for 5 minute and compression dressing was applied for 24 hour.

**Figure 4:**
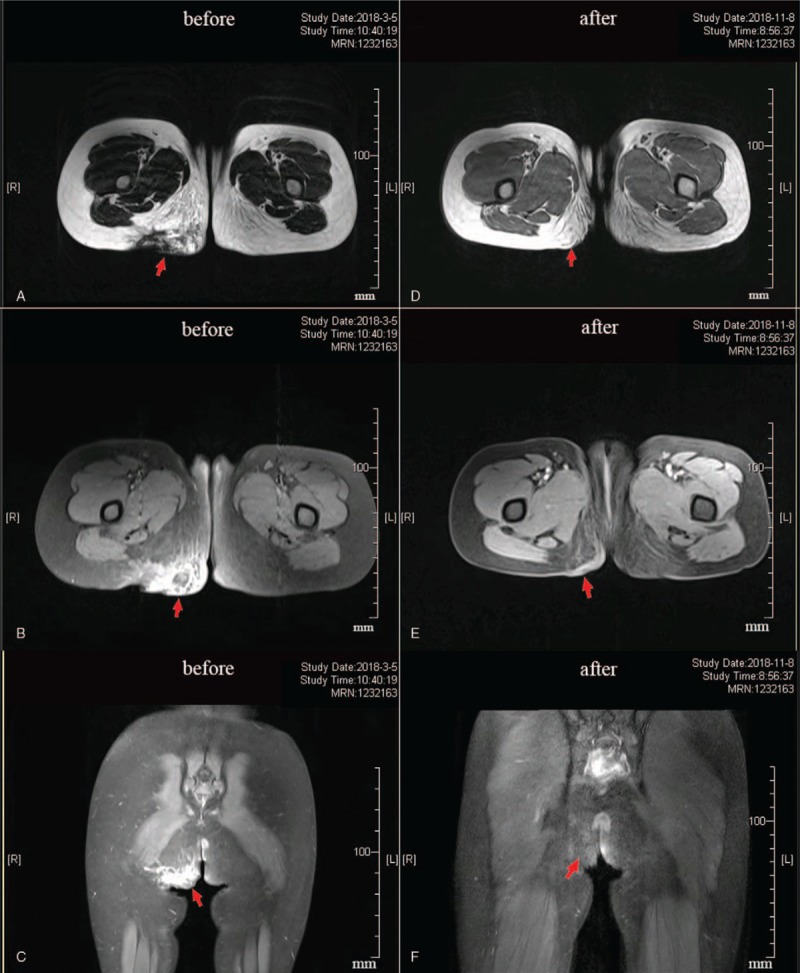
Doppler ultrasonography after treatment: Subcutaneous strip-like anechoic dark area (arrow), with clear boundary, about 29 mm in length, no blood flow signal was detected by CDFI (color Doppler flow imaging). Combined with the medical history, local subcutaneous effusion after hemangioma treatment was considered. (**A**) Slitting, (**B**) crosscut.

During the period of 8-month treatment (once a month), the mass gradually shrank and eventually disappeared. The patient underwent ultrasonography (Fig. 4) and MRI examination (Fig. 3D–F) and no suspicious lesions were seen. Clinical cure was achieved. At the 3-month follow-up, the patient was free of complications and recurrence, leading a normal life.

## 3 Discussion

Mulliken (1988) classified vascular anomalies into two major categories: hemangiomas and malformations.^[5]^ Cavernous hemangiomas, classified as venous malformations (VMs) in the slow-flow lesion category by International Society for the Study of Vascular Anomalies (ISSVA) in 2007, are not true vascular tumor. Cavernous hemangiomas have been reported to arise at all sites that head and neck are most common while hip is infrequent.^[1,2,6]^

Cavernous hemangiomas may be locally destructive because pressure is exerted on neighboring structures.^[7]^ The diversity of treatments advocated includes surgery, sclerotherapy, cautery, laser beam therapy, cryotherapy, copper needle indwelling, electrochemical, limb compression, interferon alpha-2a, steroid, cytotoxic drugs, propranolol and prophylactic antibiotic therapy.^[2,8–14]^ Among them, surgery and sclerotherapy are the most commonly used treatments at present. It is generally concluded that excisional surgery is the preferred treatment for small lesions while intralesional sclerotherapy is a cost-effective, viable method, and easy-to-perform procedure.^[14,15]^ However, these treatment modalities that may cause complications are not always satisfactory. Surgical resection carries the risks of intraoperative profuse bleeding, postoperative recurrence, long-term scarring and other complications. Major complications of sclerotherapy include hemolysis, renal toxicity, pulmonary embolism, ocular disturbances, anaphylactic reactions, hypotension, bradyarrhythmia, cutaneous necrosis, ulceration, and hyper pigmentation.^[2,13,15]^

Glucocorticoid is also one of the commonly-used and effective methods for the treatment of cavernous hemangiomas. High dose therapy in long-term systemic corticosteroids can lead to cushingoid features, low blood potassium, osteoporosis, peptic ulcer, and aggravation of infection.^[16]^ However, intralesional injection of TCA avoids the influence of long-term and massive use of corticosteroids on the growth and development of infants, with less adverse reactions, more efficiency and operation convenience/simplicity. TCA whose therapeutic mechanism is not fully understood is a new type of glucocorticoid synthesized artificially. TCA is a kind of suspension agent which contrasts with other high-potency steroids such as hydrocortisone in that TCA contains an acetonide ester that decreases its solubility at the site of injection resulting in delayed elimination,^[4]^ when it is injected into the lesion, its deposits may be located in the stroma of cavernous hemangioma or in the vascular sinus. When the deposit is located in the stroma, it has a squeezing effect on the vascular sinus or microvascular cavity. When the deposit is located in the vascular sinus, it may partially block the vascular sinus or induce platelet adhesion locally and micro-thrombosis, resulting in vascular occlusion. Thus, it can be seen that the solid deposits of TCA may play a drug-induced “vasoligation” effect on cavernous hemangioma, which can also prolong the drug's residence time and cause fibrosis of the lesion. Therefore, the possible mechanism of TCA in the treatment of cavernous hemangiomas is the combination of mechanical embolization and the effect of glucocorticoid.

A therapeutic reference standard for treatment is still inadequate.^[15]^ We should choose the most suitable treatment according to the actual situation of the patient (lesion location, size and evolution stage, and the patient's age) because there is no perfect therapy.^[16]^

In our case, we tried to not only improve clinical efficacy but also reduce the drug's possible side effects. The girl relapsed only a month after surgery. Reoperation was obviously unsuitable for her, with the wide range of lesions and the high risk of recurrence. Sclerotherapy is at high risk due to its serious complications. Propranolol is suitable for treating strawberry hemangiomas but not effective for cavernous hemangiomas.^[12]^ Therefore, we adopted ultrasound-guided subcutaneous injection of TCA, instead of a systemic high dose of hormone therapy. TCA injection we used is a kind of mixed suspension which not only acts as a hormone, but may also have a short-term vascular embolization effect.

In conclusion, Cavernous hemangiomas referred to as venous malformations (VMs), the treatment of cavernous hemangiomas is controversial. That's why this paper recommends intralesional injection of TCA for treating cavernous hemangiomas. This treatment may be a more effective and safer choice for cavernous hemangiomas.

## Author contributions

**Data curation:** Luan-hong Wang.

**Methodology:** Zhen-ying Lin.

**Supervision:** Guang Huang.

**Writing – original draft:** Wen-yan Wang.

**Writing – review & editing:** Han Lin.

Han Lin orcid: 0000-0002-7069-4787.
